# Supplementary material for: Predicting youth diabetes risk using NHANES data and machine learning
Source: Sci Rep. 2021 May 27;11:11212. doi: 10.1038/s41598-021-90406-0 (PMC8160335; doi:10.1038/s41598-021-90406-0)
Supplement: Supplementary file 1 — Supplementary Information. [file 41598_2021_90406_MOESM1_ESM.pdf]

Supplemental Information

Predicting youth diabetes risk using NHANES data and machine learning

Nita Vangeepuram, MD, MPH, Bian Liu, PhD, Po-hsiang Chiu, PhD, Linhua Wang, MS, Gaurav Pandey, PhD

Details of ML methods used for predicting preDM/DM status

The goal of ML-based classification methods[1] is to automatically infer a mathematical function that can categorize individuals, described in terms of relevant *features*, into classes, here preDM/DM or not. The classification methods we considered for this purpose were AdaBoost(M1), LogitBoost, Naïve Bayes, Logistic (Regression), Support Vector Machine (SMO), Voted Perceptron, K-nearest neighbor (IBk), PART and J48 decision tree inference algorithms, and Random Forest[1]. Their implementations in the Weka ML toolkit[2], incorporated into our DataSink pipeline[3], were used with their default parameter values in Weka. Since these implementations produce probabilistic predictions, we used the value of the probability score that yields the highest value of balanced accuracy, the mean of specificity and sensitivity[4], as the threshold to convert them into binary predictions to be comparable with the screening test results. All these ML algorithms were tested in a five-fold cross-validation (CV) setup[5], where in each CV round, 80% of the data were used to train candidate classifiers, which were then used to make preDM/DM predictions on the remaining 20% of the data. To address the imbalance between the preDM/DM and non-preDM/DM classes in the data (**Table 3**), which can adversely affect the classifiers[6], our framework balanced the two classes by randomly under-sampling the majority class during training[6], while retaining the distribution in the test fold exactly the same. Repeating this process over the whole dataset, and collecting the predictions for all five subsets of the data created during CV, generated dataset-wide predictions from all the algorithms, which were then evaluated as described below.

Details of evaluation measures used to assess preDM/DM classifications

Although several measures are known for evaluating preDM/DM classifications against true labels,[7] determined here based on ADA biomarker measurements, the most commonly used measures like accuracy and AUC score are not ideal for our data. For instance, since only about 30% of the youth in the NHANES population considered in our study are preDM/DM cases (**Table 3**), one can get an artificially high accuracy value of about 70% just by classifying all the individuals as not having preDM/DM, which is obviously not useful. The AUC score, which is designed for continuous predictions, not binary ones, and thus isn't directly applicable in our case, is also not informative in cases of class imbalance like ours.[8]

Based on these considerations, we focused on evaluation measures that have been designed for and shown to be the most relevant for classifier evaluation in the case of unbalanced classes.[8] Specifically, we used six the following measures to evaluate the correctness of the preDM/DM predictions from the screening guidelines and ML methods[7]: sensitivity (recall+), specificity (recall-), positive predictive value (PPV, precision+), negative predictive value (NPV, precision-), and F-measures for the two classes (see **Table 3** and **Supplementary Figure S1** for detailed definitions of these measures). Another advantage of these measures, as compared

to accuracy and AUC score, is that they provide per-class performance assessment, which can reveal if the classifier is biased in favor of a particular class, which is likely in the case of unbalanced classes.[8] Thus, the use of these measures provides a comprehensive assessment of the preDM/DM screening guidelines and classifiers evaluated in our work.

	PREDICTED CLASS		
ACTUAL CLASS		Class = +	Class = –
	Class = +	a (TP)	b (FN)
	Class = –	c (FP)	d (TN)

$$Precision + (P_+) = PPV = \frac{a}{a+c} \qquad Precision - (P_-) = NPV = \frac{d}{b+d}$$

$$Recall + (R_+) = Sensitivity = \frac{a}{a+b} \qquad Recall + (R_+) = Specificity = \frac{d}{c+d}$$

$$F - measure + (F_+) = \frac{2P_+R_+}{P_+ + R_+} = \frac{2a}{2a+b+c}$$

$$F - measure - (F_-) = \frac{2P_-R_-}{P_- + R_-} = \frac{2d}{2d+b+c}$$

Supplementary Figure S1. Evaluation measures for preDM/DM classifiers that generate a + or - prediction for a given individual. The relationships between sensitivity, specificity, positive/negative predictive values, precision, recall and F-measure, the evaluation measures used in our work, are summarized here. F-measure, which is a harmonic (conservative) mean of precision and recall that is computed separately for each class, provides a comprehensive and reliable assessment of model performance when classes are imbalanced, as is the case with preDM/DM classification.

Evaluation of clinical screening guidelines on weighted NHANES data

To assess the robustness of the results presented in the main text, we also evaluated the performance of the clinical screening guidelines on the population-weighted version of NHANES data. The weighted population characteristics are shown in **Supplementary Table S1**, and the guideline performance results obtained are detailed in **Supplementary Table S2** and **Supplementary Figure S2**.

Supplementary Table S1. Population-weighted characteristics of the study population (weighted sample n=32,096,599). Results based on survey procedures that took into account of the NHANES survey design.

	**Weighted %**	**Normal --- Weighted %**	**PreDM/DM --- Weighted %**
**Total**	100	72.42	27.58
**Sex**			
Male	50.9	64.53	35.47
Female	49.1	80.60	19.4
**Race/Ethnicity**			
Non-Hispanic white	57.8	75.07	24.93
Non-Hispanic black	14.7	70.57	29.43
Hispanic	19.9	66.65	33.35
other	7.6	70.89	29.11
**Age group**			
12-14 years	36.71	68.64	31.36
15-17 years	40.13	76.41	23.59
18-19 years	23.17	71.48	28.52
**BMI percentile categories**			
BMI $<85^{th}$	62.35	75.43	24.57
$85^{th} \leq$ BMI $< 95^{th}$	16.26	76.90	23.1
$95^{th} \leq$ BMI $< 99^{th}$	15.33	65.22	34.78
BMI $\geq 99^{th}$	6.06	47.59	52.41
**At risk for preDM/DM based on AAP/ADA pediatric clinical screening guidelines**			
No	71.18	74.05	25.95
Yes	28.82	63.66	36.34
	Weighted Mean (SE; Median)	Weighted Mean (SE; Median)	Weighted Mean (SE; Median)
**Age (years)**	15.5 (0.06; 15.0)	15.5 (0.10; 15.1)	15.3 (0.10; 14.7)
**Fasting plasma glucose (FPG, mg/dL)**	94.1 (0.26; 93.8)	91.4 (0.19; 91.8)	101.4 (0.36; 102)
**Two hour plasma glucose (2hrPG, mg/dL)**	97.5 (0.61; 94.4)	92.4 (0.57; 91.1)	110.8 (1.25; 105)
**Hemoglobin A1c (HbA1c, %)**	5.2 (0.01; 5.1)	5.1 (0.01; 5.1)	5.3 (0.02; 5.3)
**Cholesterol, total (mg/dL)**	159.1 (0.76; 155.6)	158.4 (0.95; 153.7)	161.1 (1.52; 158)
**BMI-for-age percentile**	67.1 (0.71; 75.1)	66.0 (0.77; 73.2)	69.9 (1.70; 78.8)

Note: ADA: American Diabetes Association; AAP: American Academy of Pediatrics; preDM: prediabetes; DM: diabetes; BMI: body mass index. SE: standard error.

Supplementary Table S2. Population-weighted performance measures of pediatric clinical screening guideline when compared against prediabetes/diabetes (preDM/DM) determinations based on biomarker criteria. Results based on survey procedures that took into account of the NHANES survey design.

		**AAP/ADA pediatric clinical screening guidelines**		
**preDM/DM based on elevated FPG/2hrPG/HbA1C**		**Yes**	**No**	*Row total*
	**Yes**	**3,217,479**	**5,635,968**	8,853,447
	**No**	**6,032,038**	**17,211,114**	23,243,152
	Column total	*9,249,517*	*22,847,082*	32,096,599
**Performance measures of the adult screener/pediatric clinical screening guidelines when compared against preDM/DM based on biomarkers for the positive (+) and negative class (-)**				
**Sensitivity (recall+)** = Proportion of at-risk based on pediatric clinical screening guidelines that have preDM/DM based on biomarkers		3,217,479/8,853,447 = 36.34%		
**Specificity (recall-)** = Proportion of not at-risk based on pediatric clinical screening guidelines that do not have preDM/DM based on biomarkers		17,211,114/23,243,152 = 74.05%		
**Positive Predictive Value (PPV, precision+)** = Proportion of youth identified with preDM/DM based on biomarkers among all predicted to be at-risk based on pediatric clinical screening guidelines		3,217,479/9,249,517 = 34.8%		
**Negative Predictive Value (NPV, precision-)** = Proportion of youth not identified with preDM/DM based on biomarkers among all predicted not to be at-risk based on pediatric clinical screening guidelines		17,211,114/22,847,082 = 75.33%		
**F-measure+** = Harmonic (conservative) mean of Precision+ and Recall+ = 2*(Precision+ ***** Recall+) / (Precision+ **+** Recall+)		2*(36.34%*34.8%)/(36.34%+34.8%) = 35.55%		
**F-measure-** = Harmonic (conservative) mean of Precision- and Recall- = 2*(Precision- ***** Recall-) / (Precision- **+** Recall-)		2*(74.05%*75.3%) 74.05%+75.3%) = 74.68%		

Note: ADA: American Diabetes Association; AAP: American Academy of Pediatrics; preDM: prediabetes; DM: diabetes; BMI: body mass index; FPG: fasting plasma glucose; 2hrPG: 2 hour plasma glucose; HbA1c: hemoglobin A1c.

Supplementary Figure S2. Population-weighted variations in the performance of the American Diabetes Association pediatric screening guidelines in identifying youth with prediabetes/diabetes (preDM/DM) based on biomarker measurements across subgroups stratified by age group (12-14, 15-17, and 18-19), race/ethnicity (Hispanic, non-Hispanic Black, non-Hispanic white, other), and sex (female, male). Horizontal red lines denote the value of the corresponding evaluation measure obtained from the full weighted study population (youth ages 12-19, National Health and Nutrition Examination Survey data, 2005-2016). Results based on survey procedures that took into account of the NHANES survey design.

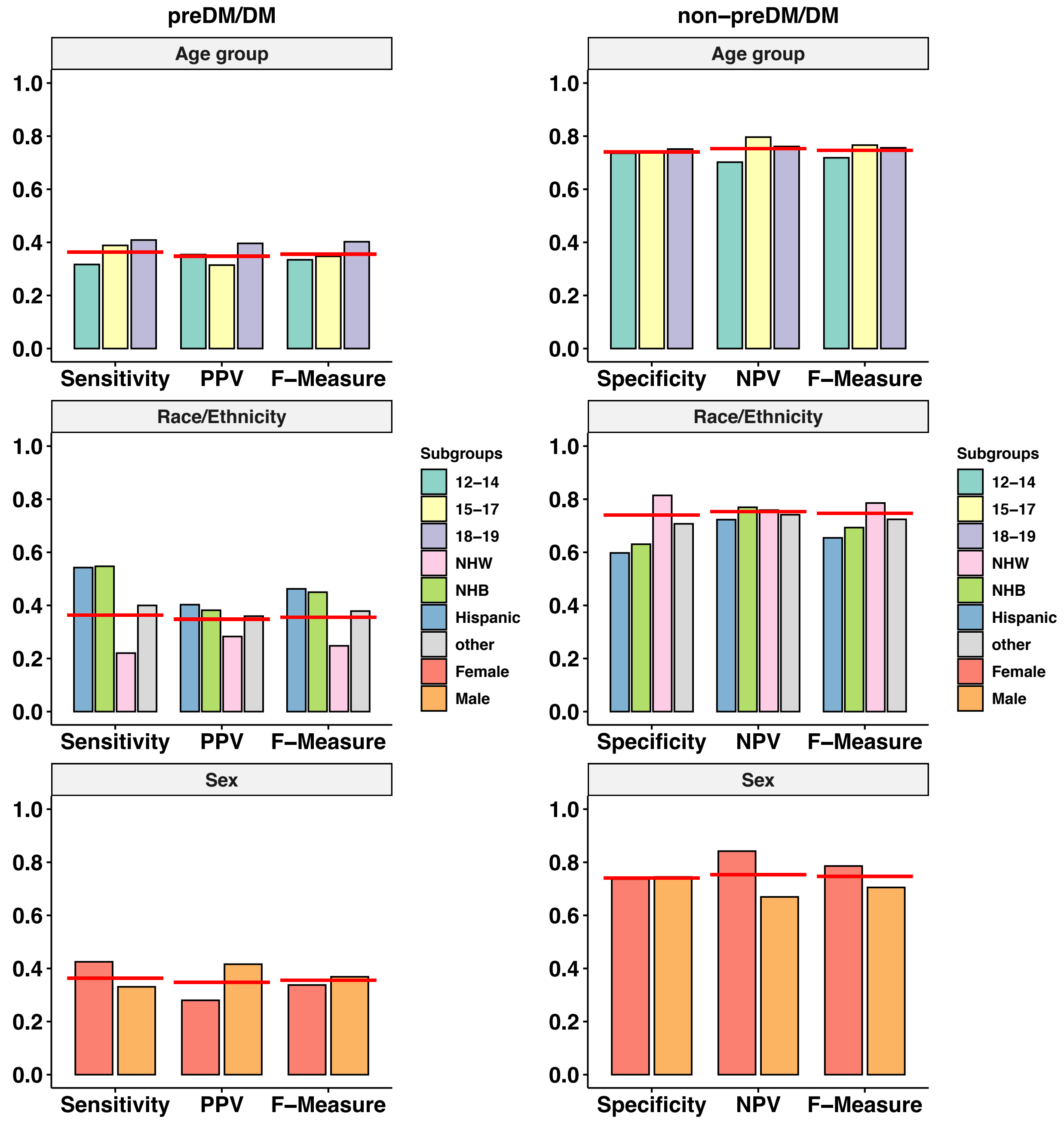

References

1. Alpaydin E. *Introduction to machine learning.* MIT press; 2014.
2. Hall M, Frank E, Holmes G, Pfahringer B, Reutemann P, Witten IH. The WEKA data mining software: an update. *SIGKDD Explor Newsl.* 2009;11(1):10-18.
3. Whalen S, Pandey OP, Pandey G. Predicting protein function and other biomedical characteristics with heterogeneous ensembles. *Methods.* 2016;93:92-102.
4. Sokolova M, Japkowicz N, Szpakowicz S. Beyond accuracy, F-score and ROC: a family of discriminant measures for performance evaluation. Paper presented at: Australasian joint conference on artificial intelligence2006.
5. Arlot S, Celisse A. A survey of cross-validation procedures for model selection. *Statist Surv.* 2010;4:40-79.
6. Chawla NV. Data Mining for Imbalanced Datasets: An Overview. In: Maimon O, Rokach L, eds. *Data Mining and Knowledge Discovery Handbook.* Boston, MA: Springer US; 2005:853-867.
7. Lever J, Krzywinski M, Altman N. Points of Significance: Classification evaluation. *Nat Meth.* 2016;13(8):603-604.
8. Saito T, Rehmsmeier M. The precision-recall plot is more informative than the ROC plot when evaluating binary classifiers on imbalanced datasets. *PLoS One.* 2015;10(3):e0118432.
